# Control programs for strongyloidiasis in areas of high endemicity: an economic analysis of different approaches

**DOI:** 10.1186/s40249-021-00858-9

**Published:** 2021-05-25

**Authors:** Dora Buonfrate, Lorenzo Zammarchi, Zeno Bisoffi, Antonio Montresor, Sara Boccalini

**Affiliations:** 1 Department of Infectious Tropical Diseases and Microbiology, IRCCS Sacro Cuore Don Calabria Hospital, Negrar, Verona Italy; 2 Department of Experimental and Clinical Medicine, University of Florence, Florence, Italy; 3 Referral Center for Tropical Diseases of Tuscany, Infectious and Tropical Diseases Unit, Careggi University Hospital, Florence, Italy; 4 Department of Diagnostics and Public Health, University of Verona, Verona, Italy; 5 Department of Control of Neglected Tropical Diseases, World Health Organization, Geneva, Switzerland; 6 Department of Health Sciences, University of Florence, Florence, Italy

**Keywords:** *Strongyloides stercoralis*, Strongyloidiasis, Control programme, Preventive chemotherapy, Ivermectin, Economic, Adverted death, Adverted infection

## Abstract

**Background:**

Implementation of control programmes for *Strongyloides stercoralis* infection is among the targets of the World Health Organization Roadmap to 2030. Aim of this work was to evaluate the possible impact in terms of economic resources and health status of two different strategies of preventive chemotherapy (PC) compared to the current situation (strategy A, no PC): administration of ivermectin to school-age children (SAC) and adults (strategy B) versus ivermectin to SAC only (strategy C).

**Methods:**

The study was conducted at the IRCCS Sacro Cuore Don Calabria hospital, Negrar di Valpolicella, Verona, Italy, at the University of Florence, Italy, and at the WHO, Geneva, Switzerland, from May 2020 to April 2021. Data for the model were extracted from literature. A mathematical model was developed in Microsoft Excel to assess the impact of strategies B and C in a standard population of 1 million subjects living in a strongyloidiasis endemic area. In a case base scenario, 15% prevalence of strongyloidiasis was considered; the 3 strategies were then evaluated at different thresholds of prevalence, ranging from 5 to 20%. The results were reported as number of infected subjects, deaths, costs, and Incremental-Effectiveness Ratio (ICER). A 1-year and a 10-year horizons were considered.

**Results:**

In the case base scenario, cases of infections would reduce dramatically in the first year of implementation of PC with both strategy B and C: from 172 500 cases to 77 040 following strategy B and 146 700 following strategy C. The additional cost per recovered person was United States Dollar (USD) 2.83 and USD 1.13 in strategy B and C, respectively, compared to no treatment in the first year. For both strategies, there was a downtrend in costs per recovered person with increasing prevalence. The number of adverted deaths was larger for strategy B than C, but cost to advert one death was lower for strategy C than B.

**Conclusions:**

This analysis permits to estimate the impact of two PC strategies for the control of strongyloidiasis in terms of costs and adverted infections/deaths. This could represent a basis on which each endemic country can evaluate which strategy can be implemented, based on available funds and national health priorities.

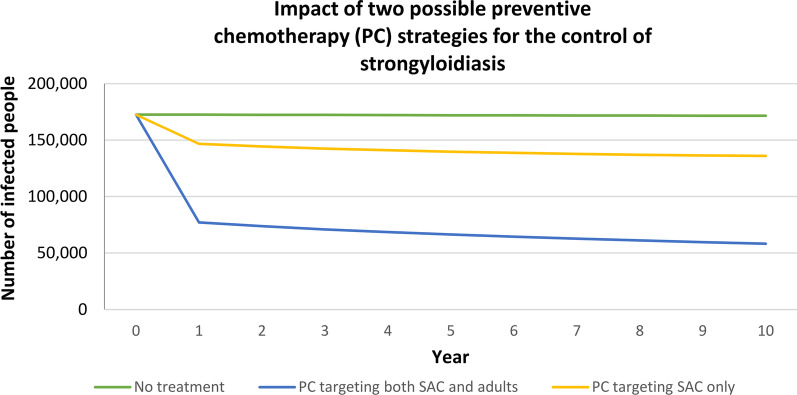

## Background

The soil-transmitted helminth (STH) *Strongyloides stercoralis* causes relevant morbidity in affected population, and can cause the death of infected people in case of immunosuppression [1]. According to recent estimates, around 600 million people are affected worldwide, with most cases distributed in South East Asia, Africa, and the Western Pacific Region [2]. Based on the recent evidence on the global burden of strongyloidiasis, the World Health Organization (WHO) has included the control of *S. stercoralis* infection among the targets of the Neglected Tropical Diseases (NTD) Roadmap to 2030 [3]. This is the first time that the WHO recommends a control program for strongyloidiasis, and a specific control approach is under definition.

*S. stercoralis* shares the route of transmission with hookworm and has similar geographical distribution to the other STH, but needs a different diagnostic approach and treatment [4]. Indeed, Kato-Katz, which is used to assess the prevalence of STH in control programs, has exceedingly low sensitivity for *S. stercoralis*. There are other diagnostic methods with higher accuracy that can be recommended for this parasite: Baermann and agar plate culture among the parasitological methods, polymerase chain reaction, and serological assays [5]. The latter methods are used for other NTD, taking advantage from the possibility of collection of blood on filter paper, which allows a rapid collection and an easy storage of the biological samples [6, 7].

Unfortunately there is no gold standard for the diagnosis of this parasite [5], hence the choice of the best diagnostic approach for deployment in control programs should take into consideration several factors such as accuracy of the test, cost and feasibility for use in the field. In a recent meeting organized by the WHO [8], selected experts identified the serological assessment as the best available option and the NIE ELISA as the best choice among the commercially available ELISA kits. As for treatment, preventive chemotherapy (PC) for STH entails the administration of a benzimidazole drug, either albendazole or mebendazole [3]. These programs often target school-age children (SAC), who present the highest clinical burden caused by STH [3]. However, benzimidazoles have scarce efficacy against *S. stercoralis*, for which ivermectin is the drug of choice [9]. Ivermectin has instead been used for decades for mass treatment in the context of the elimination programs for onchocerciasis and lymphatic filariasis (NTD) [10, 11]. It has excellent safety profile and tolerability, but it is not recommended for children younger than 5 years of age [12].

*S. stercoralis* differs from the other STH also in terms of duration of the infection, as a peculiar auto-infective cycle causes an indefinite persistence of the parasite in the human host, if not adequately treated. This also leads to a higher in prevalence of infection in adulthood, as a result of new infections over time and persistence of long-term disease [1, 2].

Despite the peculiarities, the implementation of a control program for strongyloidiasis might benefit from the integration of specific activities with the already existing programs for other NTDs. Sharing infrastructures and staff might result in lower costs and a more rapid onset of activities aimed at the control of *S. stercoralis.*

The aim of this work is to estimate costs and outcomes resulting from different strategies relating the control of strongyloidiasis, namely: (A) no intervention; (B) mass drug administration targeting SAC and adults; (C) PC targeting SAC.

## Material and methods

### Study design and strategies

**Fig. 1:**
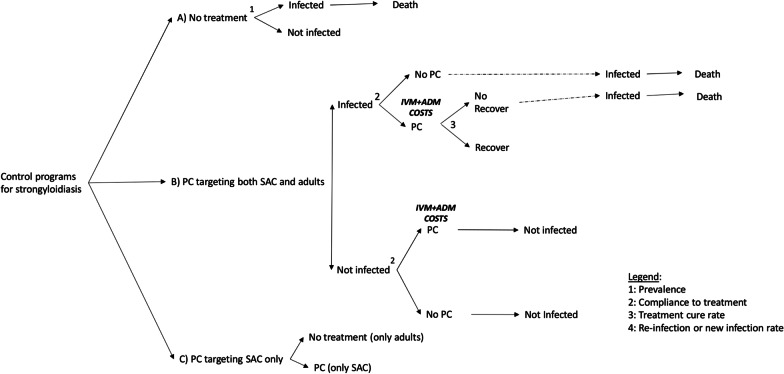
Decisional tree of health status. *PC* preventive chemotherapy, *IVM* ivermectin, *ADM* administration, *SAC* school-age children

The study was conducted at the IRCCS Sacro Cuore Don Calabria hospital, Negrar di Valpolicella, Verona, Italy, at the University of Florence, Italy, and at the WHO, Geneva, Switzerland, from May 2020 to April 2021. Data source for the model was available literature. A mathematical model was developed in Microsoft® Excel® for Microsoft 365 MSO (Microsoft Corporation, Santa Rosa, California, USA) to assess the clinical and economic impact of two possible interventions for strongyloidiasis in areas of high endemicity compared with (A) no intervention (current practice); (B) PC targeting both SAC and adults; (C) PC targeting SAC only. One and 10-year time-horizon were evaluated in the analysis. The study was conducted according to the perspective of the local National Health System, which is in charge of deworming programs, including associated direct costs from the public sector of financing. The decisional tree and data input are reported in Fig. 1 and Table 1, respectively. Particularly, the decisional tree shows the mutually exclusive health statuses foreseen by the model and the logical steps of calculations for each different strategy. The transition rate from one state to the next one and the related assumptions are reported in detail in the following input data section. The results are reported as number of infected subjects, not infected subjects, cured subjects (recovered), deaths, costs, and Incremental Cost-Effectiveness Ratio (ICER) which is the difference in costs between two strategies divided by the difference in their effects as recovered subjects and averted infections. The smaller ICER indicates better cost-effectiveness of one strategy versus the other.

**Table 1:** Data input of the mathematical model for the case base scenario

Population data	%	Number	–	Refs
Total population		1 000 000		
School age population (6–14 years)	0.25	250 000		[13]
Adult population (≥ 15 year)	0.50	500 000		[13]
School age (6–14 years) participating to the survey	0.001	1000		*
Adults (≥ 15 year) participating to the survey	0.000			*

*Strongyloides stercoralis* infection and treatment data	Case base scenario (%)	Min.	Max.	Refs
Strongyloidiasis prevalence in school age population (6–14 years)	0.150	0.10	0.20	[2]
Strongyloidiasis prevalence in adults (≥ 15 year) estimated with the survey	0.270	0.18	0.36	[2]
Compliance to ivermectin treatment in school age group (6–14 years)	0.80	0.75	0.85	[14]
Compliance to ivermectin treatment in adults (≥ 15 year)	0.60	0.55	0.65	[14]
Treatment cure rate (is the same in all age groups)	0.86	0.79	0.91	[15]
Re-infection or new infection rate in school age group (6–14 years)	0.50	0.45	0.55	[16]
Re-infection or new infection rate in adults (≥ 15 year)	0.50	0.45	0.55	[16]

Costs in dollars	Case base scenario	Min.	Max.	Refs
Cost of survey per person (Baerman / ELISA + additional costs such as logistics etc.…)	27.00			§
Cost of ivermectin for 1 school age child	0.10	0.05	0.20	[21]
Cost of ivermectin for 1 adult	0.30	0.20	0.40	[21]
Cost of ivermectin administration at school	0.015	0.01	0.02	[20]
Cost of ivermectin administration to adults	0.50	0.25	0.75	[19]

*Based on expert’s opinion. § Buonfrate D. personal communication from a pilot project in Ethiopia

### Data input

We assumed a standard population of 1 000 000 subjects living in a country highly endemic for strongyloidiasis, of which 50% adults (≥ 15 years) and 25% school age children (6–14 years). This is a distribution frequently observed in South East Asia, Africa, and the Western Pacific Region countries [13]. Prevalence of strongyloidiasis in the case base scenario was estimated to be 27% and 15% in adults and SAC, respectively [2].

In strategy A (current practice) subjects receive no treatment, so we assumed that, at the end of both the 1 and 10-year-periods, the prevalence of the infection would remain unaltered.

In strategy B, both SAC and adults would be offered PC. According to a compliance rate estimated to be 60% in adults and 80% in SAC [14], both infected and uninfected subjects would receive a single dose of ivermectin once a year for 10 years. We assume a cure rate for infected subjects of about 86% [15]. As the community would continue to be exposed to the source of infection (although the contamination of the soil would presumably decrease over time since the beginning of the PC), re-infections and new infections would continue to occur. The yearly rate of new infections is estimated as half of the baseline infection prevalence [16]. Therefore, from the second year of PC implementation onwards, each year the number of infected cases will be equal to the sum of new infections plus cases remaining positive (i.e. people who did not receive PC and those who did not respond to treatment). The strategy C (PC targeting SAC only) is similar to B, with the only difference that only SAC would receive ivermectin while adults would not.

In all strategies, the estimated number of deaths due to severe strongyloidiasis were subtracted each year from the population. These deaths were estimated assuming that 0.4% of infected subjects would develop severe strongyloidiasis [17], and 64.25% of them would die [18]. Deaths due to other causes were not included in the model.

The impact of the two strategies was then evaluated at different levels of prevalence of strongyloidiasis in SAC: 5% (corresponding to 9% prevalence in adults), 10% (18%), and 20% (36%).

### Costs

We assumed that strategy A is not associated with any direct cost for the National Health System, even though strongyloidiasis-related morbidity would have a possible, though presumably marginal, economic impact on the health system due to hospitalization and outpatient consultation. The advantages from the societal point of view (sucha as increase in productivity and school attendance, and decrease of time lost for consultation) although probably relevant were not taken into consideration because of the difficulties in estimating them precisely.

For the implementation of strategies B and C we considered several costs. First step would be the conduction of a survey involving 0.1% of the SAC population in order to ascertain the prevalence of the infection in the selected area. The survey would have a cost of United States Dollar (USD) 27 per subject, including costs for parasitological (Baermann) and serological tests (ELISA); additional costs for logistics were partially based on a pilot project planned in Ethiopia. Overall, a survey on 250 children (0.1% of children comprised in our standard population) would cost USD 6750. The cost for ivermectin treatment for SAC and adults (USD 0.1 and USD 0.3, respectively) were based on expected cost of generic ivermectin prequalified by WHO [8]. Finally, the cost for the administration of ivermectin to SAC and adults was USD 0.015 and USD 0.5, respectively) [19, 20].

## Results

### Impact of the different strategies in terms of infection prevalence

Table 2 and Table 3 show the total number of infected and non-infected children and adults of the standard population of individuals aged over 6 years in the three strategies and related costs in the 1-year and 10-year analyses, respectively, calculated by the mathematical model. In particular, Table 2 reports the difference in the number of infected individuals due to the two PC strategies compared to the comparator (no treatment strategy). There are 172 500 infected people in the population when the prevalence is equal to 15% in children and 27% in adults. The number of infected subjects shows a 55.3% reduction introducing PC targeting both SAC and adults, and a 15% reduction in case PC targets SAC only.

**Table 2:** Estimates of infected and not infected subjects, of costs to be sustained and of the reduction in the number of infections: comparison between current situation (no treatment) and the two PC strategies considered

	Total infected	Not infected	Costs	DELTA: reduction in infected individuals (= recovered subjects)
*No treatment*				
Children	37 500	212 500		
Adults	135 000	365 000		
Total	172 500	577 500		
				DELTA: PC targeting both SAC and adults vs no treatment
*PC targeting both SAC and adults*				*Reduction in infected individuals (= recovered subjects)*
Children	11 700	238 300	29 750	25 800
Adults	65 340	434 660	240 000	69 660
Total	77 040	672 960	269 750	95 460
				DELTA: PC targeting SAC only vs no treatment
*PC targeting SAC only*				*Reduction in infected individuals (= recovered subjects)*
Children	11 700	238 300	29 750	25 800
Adults	135 000	365 000	0	0
Total	146 700	603 300	29 750	25 800

Cost of treatment per person is equal to USD 0.36 and USD 0.04 in strategy B and C, respectively. Results of the mathematical model in the 1-year horizon of analysis. *PC* preventive chemotherapy, *SAC* school-age children

**Table 3:** Estimates of infected and not infected subjects, of costs to be sustained and of the reduction in the number of infections: comparison between current situation (no treatment) and the two PC strategies considered

	Total infected individuals	Not infected	Costs	Recovered	DELTA: reduction of infections
*No treatment*					
Children	374 350	2 121 317			
Adults	1 345 792	3 638 624			
	1 720 142	5 759 941			
					DELTA: PC targeting both SAC and adults vs no treatment
*PC targeting both SAC and adults*					*Reduction of infections*
Children	54 479	2 444 666	236 671	120 133	319 871
Adults	608 307	4 384 450	2 396 523	648 526	737 485
	662 786	6 829 116	2 633 195	768 659	1 057 356
					DELTA: PC targeting SAC only vs no treatment
*PC targeting SAC only*					*Reduction of infections*
Children	54 479	2 444 666	236 671	120 133	319 871
Adults	1 345 792	3 638 624	0	0	0
	1 400 271	6 083 290	236 671	120 133	319 871

Results of the mathematical model in the 10-year horizon of analysis. *PC* preventive chemotherapy, *SAC* School-age children

In the long-time analysis (10 years) the reduction of infections increases to 61.6% and 18.6% respectively, in strategy B and C compared to strategy A. In addition, applying strategy B and C could lead to a reduction of 61% and 48% deaths in 10 years compared to no treatment, respectively.

**Fig. 2:**
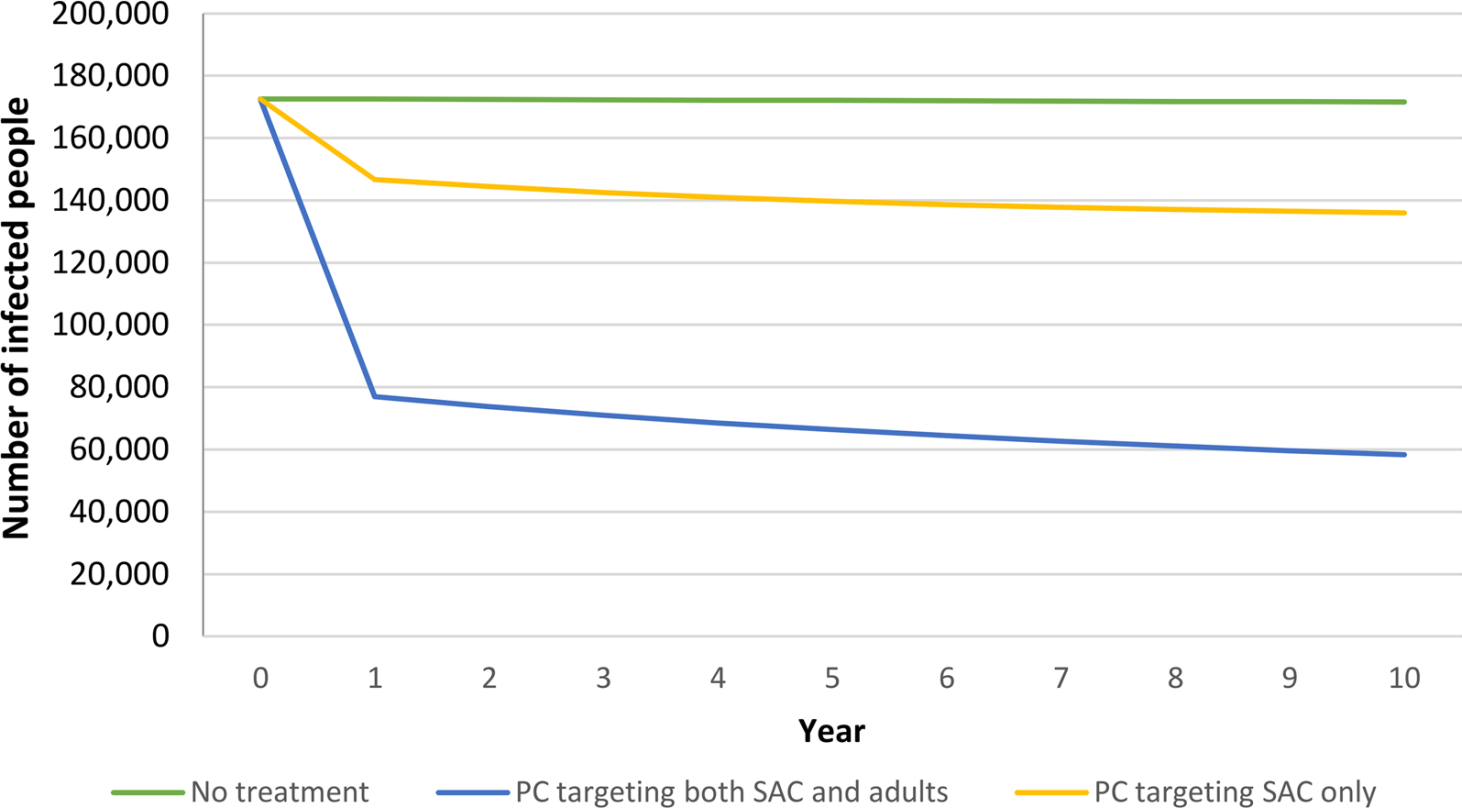
Estimates of the reduction in the number of infected people over years, according to the three strategies. *PC* preventive chemotherapy, *SAC* School-age children

Figure 2 shows the number of infected people in the three strategies during the 10-year period of analysis: while the figure remains unvaried without intervention, in the first years of implementation of both PC strategies we have a quick decrement in cases, which decrease more slowly afterwards.

### Impact of the different strategies in terms of costs

**Fig. 3:**
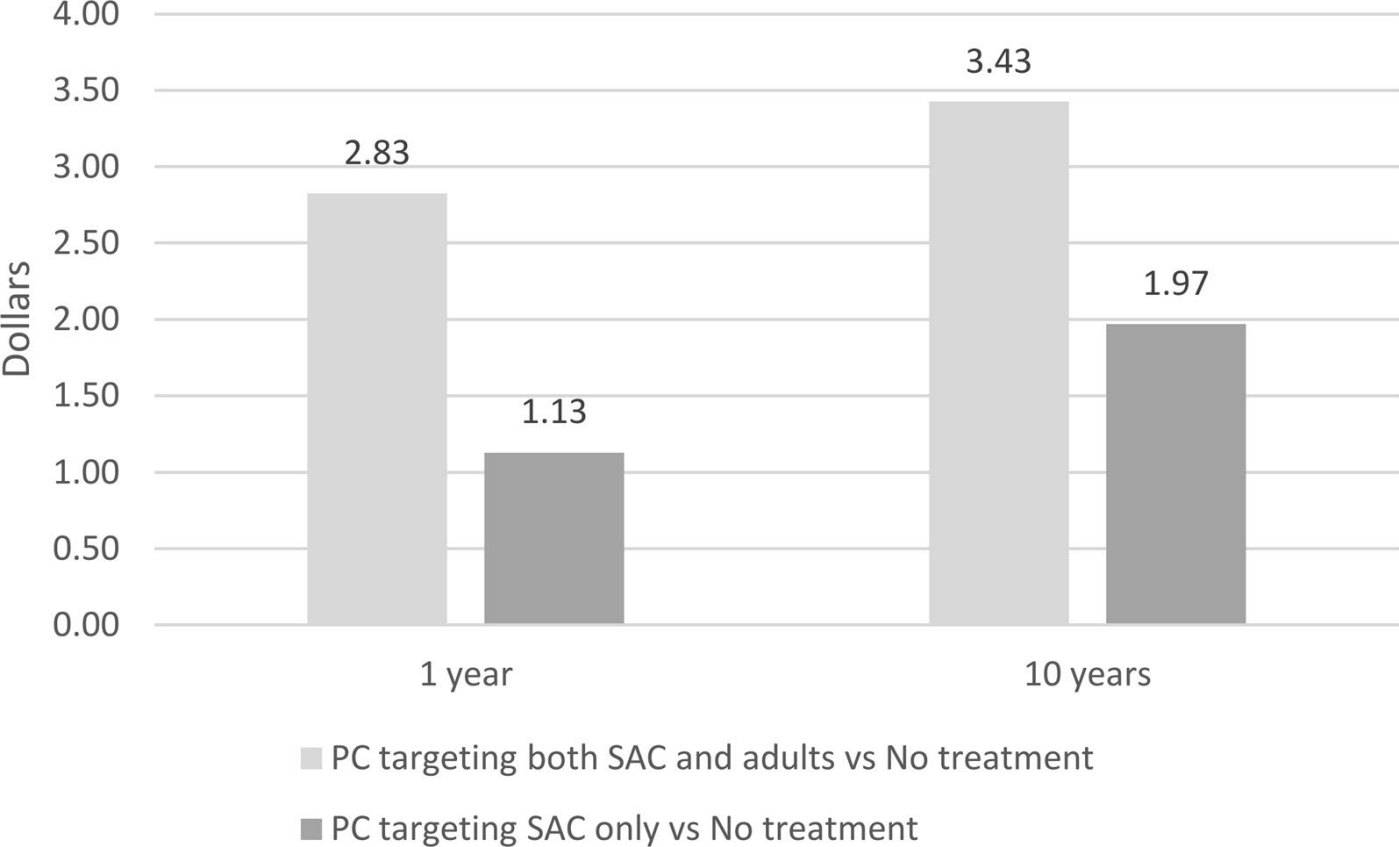
Cost per recovered person in the 1-year and 10-year analyses. *PC* preventive chemotherapy, *SAC* School-age children

Concerning the ICERs, the additional cost per recovered person increases slightly from the 1 to the 10-year analysis (Fig. 3). Considering the reduction of infected individuals in the population, in the 10-year period the cost per avoided infection is USD 2.49 and USD 0.74 in strategy B and C, respectively, compared to no treatment.

**Fig. 4:**
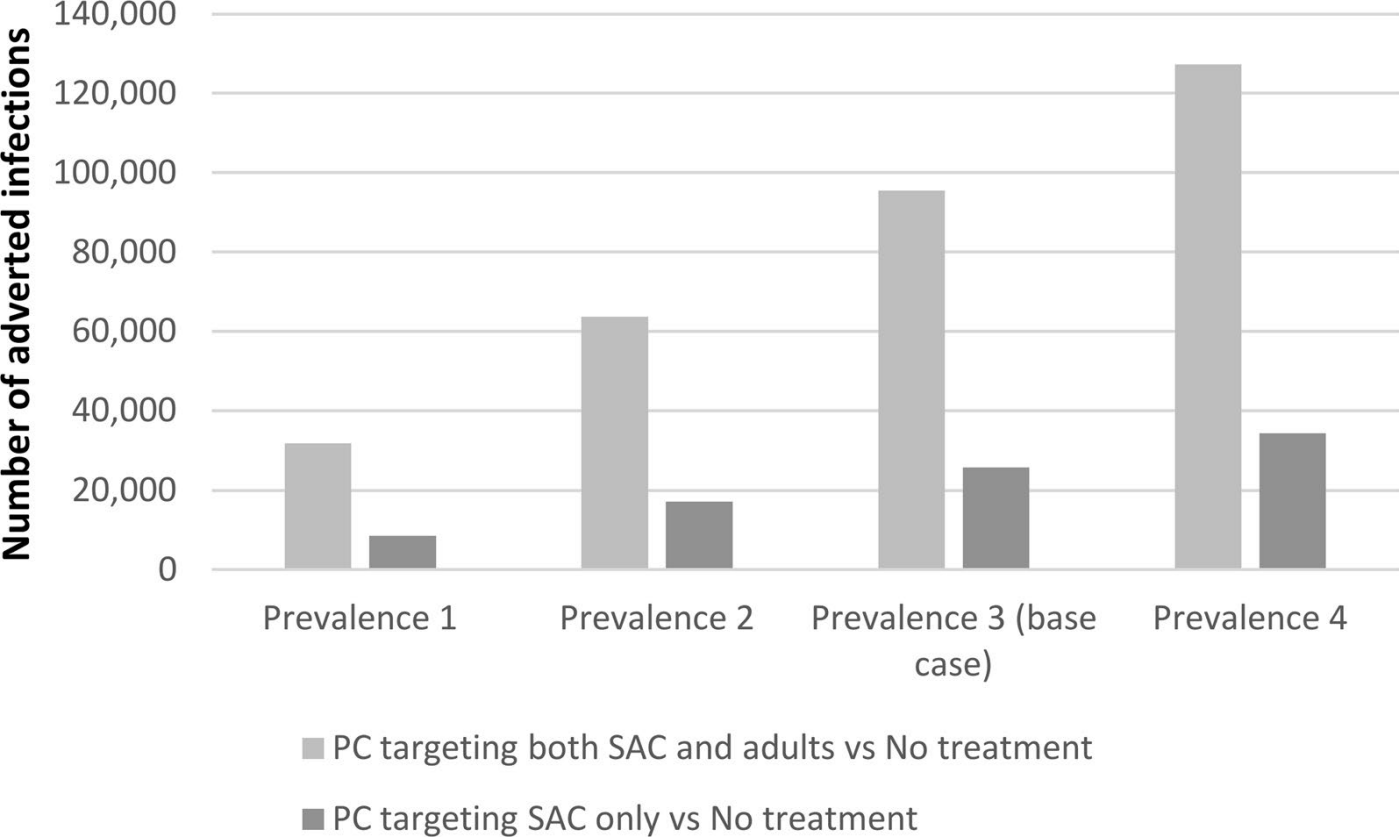
Number of adverted infections for prevalence values ranging from 5 to 20% in the first year. *PC* preventive chemotherapy, *SAC* School-age children

**Fig. 5:**
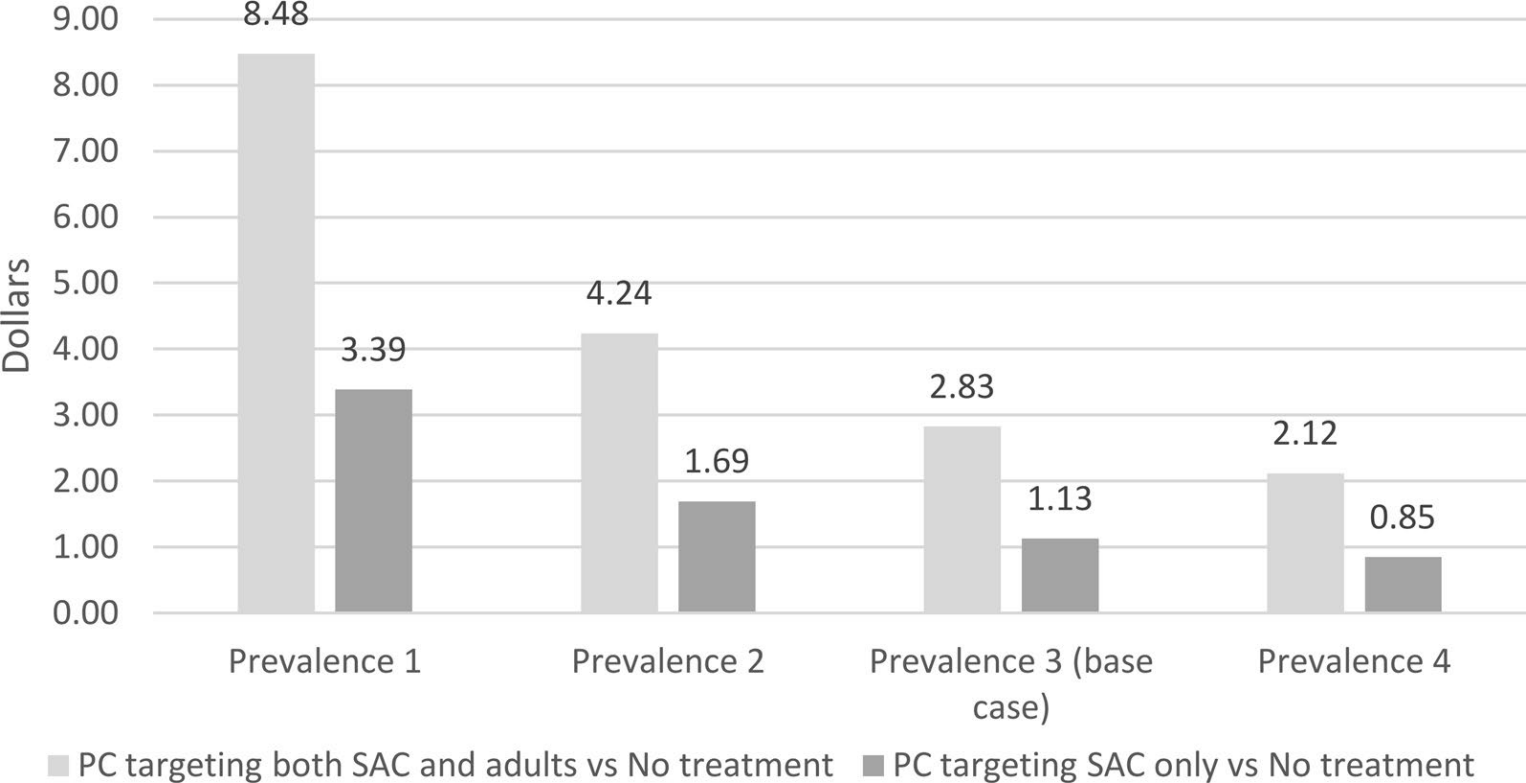
Cost per recovered person for prevalence values ranging from 5 to 20% in the first year. *PC* preventive chemotherapy, *SAC* School-age children

Figures 4 and 5 report the number of infections avoided with PC and related cost per recovered person compared to no treatment, for prevalence values ranging from 5 to 20% in the one-year horizon. Particularly, compared to the base case scenario, in settings with lower prevalence (for instance 10% in children and 18% in adults), the cost per recovered person would be higher; a lower cost would instead be needed in settings where prevalence is higher.

Number of adverted deaths and relative costs in the different PC strategies, at the 1 and 10-year horizons are resumed in Table 4. Costs to avert one death result lower for strategy C than for strategy B, for all prevalence considered. For both strategies, costs reduce over time and show a downtrend with increasing prevalence.

**Table 4:** Estimates of number of adverted infections and deaths and costs to avert one death following the different strategies, at the 1 and 10-year horizons

Strategy Horizon	Averted deaths, Number of individuals		Delta costs, in USD		Cost to avert one death, in USD		Averted infections, Number of individuals	
	B vs A	C vs A	B vs A	C vs A	B vs A	C vs A	B vs A	C vs A
Prevalence 1*								
1 year	82	22	269 750	29 750	3299	1346	31 820	8600
10 years	909	274	2 635 565	236 724	2901	863	353 531	106 768
Prevalence 2**								
1 year	164	44	269.750	29 750	1649	673	63 640	17 200
10 years	1814	548	2 634 380	236 698	1452	432	705 982	213 392
Prevalence 3 (base scenario)***								
1 year	245	66	269 750	29 750	1100	449	95 460	25 800
10 years	2717	822	2 633 195	236 671	969	288	1 057 356	319 871
Prevalence 4****								
1 year	327	88	269 750	29 750	825	337	127 280	34 400
10 years	3618	1095	2 632 010	236 645	728	216	1 407 654	426 207

*USD* United States Dollars, *A* no treatment, *B* PC targeting SAC and adults, *C* PC targeting SAC, *PC* preventive chemotherapy, *SAC* School-age children

*Prevalence 1: 5% in school aged children and 9% in adults; **prevalence 2: 10% in school aged children and 18% in adults; ***prevalence 3: 15% in school aged children and 27% in adults; ****prevalence 4: 20% in school aged children and 36% in adults

## Discussion

In this work, we evaluated two possible PC strategies, in comparison to the current lack of control programmes, for the control of strongyloidiasis in terms of costs, potential impact on prevalence of strongyloidiasis and on *S. stercoralis*-related deaths in a standard population. As a first step, a baseline assessment of prevalence is recommended, and this would cost about USD 27 per tested individual (that is a total of 6750 to test 250 children). Additional costs would then depend on the chosen strategy, which could be either (A) no implementation of a PC programme (current situation, no additional costs); (B) PC administered to the whole population (USD 0.36 per treated person); (C) or PC addressing SAC (USD 0.04 per person). Both strategies B and C would lead to a dramatic reduction in the number of infected people in the first year of implementation of the PC: in a scenario with 15% prevalence in school age population and 27% in adults, the total number of infected people would reduce from 172 500 at baseline to 77 040 and 146 700 cases after implementation of strategies B and C, respectively. Afterwards, cases would still reduce, but at a slower pace. Costs per recovered person were estimated not only in relation to the two strategies (with an obvious higher cost for the implementation of strategy B compared to C, 3.43 versus USD 1.97, respectively, in the 10-year horizon), but also in relation to the baseline prevalence. The analysis showed a downtrend in costs per recovered person with increasing prevalence, going from USD 8.48 per person for strategy B and USD 3.39 for strategy C in a scenario of 5% prevalence in SAC, to USD 2.12 and 0.85 per person for the implementation of strategy B and C, respectively, in a scenario of 20% prevalence. Finally, the impact of the two strategies was analyzed in terms of adverted deaths. Strategy B obviously led to a larger number of adverted deaths (245 and 2717 at the 1-year and 10-year horizons, respectively) than strategy C (66 and 822 at the 1-year and 10-year horizons, respectively). But another relevant aspect is cost to advert one death, which reduced over time for both strategies, and was lower for strategy C (USD 288 at the 10-year horizon) than for B (USD 969 at 10 years).

The choice of a PC strategy for the control of strongyloidiasis would be based on a combination of factors, including availability of funds, national health policy, and existing infrastructures. Each country would then have a programme tailored on its specific goals and resources. Where PC programmes for the control of STH in SAC are already in place, the integration with ivermectin could be deemed easier to be implemented at reasonable costs; worth of note, lower costs would be needed to avoid one death. On the other hand, where there are no major financial constraints, PC administered to the whole population could certainly lead to a more accentuated reduction in infections, thus total *Strongyloides*-deaths would dramatically decrease over time. Indeed, the latter strategy would be supported by the distribution of infections by *S. stercoralis* observed in the population, which follows an upward trend with increasing age, the opposite of what is observed for *Trichuris trichiura* and *Ascaris lumbricoides* [22]. However, the integration of ongoing PC programmes for STH with ivermectin has additional benefits that could be considered highly valuable besides the effect on strongyloidiasis. Indeed, the combination of ivermectin plus albendazole/mebendazole demonstrated increased efficacy against *T. trichiura* than the benzimidazoles only [23]. This could be a reason to support the combined PC in SAC against concerns about the lower prevalence in this age group compared to adults. Moreover, another approach to be considered, could be an initial programme targeting SAC, which could be then scaled up to the include adolescents and adults when possible. All age groups, included or not in other PC programmes, would also benefit of the potential effect of ivermectin against ectoparasites, including scabies [24].

Another factor that would deeply influence the cost/benefit of implementation of PC with ivermectin is the prevalence of the infection in the population. For increasing values of prevalence, the reduction of infections is more pronounced, and costs per recovered person decrease. Setting a threshold for the implementation of PC against *S. stercoralis* should take into consideration a balance between these two aspects. It must be considered that for the other STH the strong recommendation to the implementation of PC where prevalence is 20% or more, is based on a significant reduction of morbidity in the target population [3]. However, this might not be a proper goal for *S. stercoralis,* due to the risk of death of the infected subject, which persist at any intensity of infection. However, costs to sustain a PC for *S. stercoralis* even at the lower prevalence would be presumably deemed too high by most endemic countries, and a threshold for treatment set at around 15–20% of prevalence might be most adequate. Moreover, at prevalence ≥ 15%, serological tests provide a more reliable estimate than at lower prevalence, where more false positives tend to occur [21]. Another factor that should be taken in account is that mass administration of ivermectin would be challenging in *Loa loa* endemic areas due to the known risk of potentially fatal encephalopathy in patients with high microfilarial blood density [25].

Further, monitoring of drug efficacy should be implemented, in consideration of the possible emergence of resistance to ivermectin after several years of massive administration [26].

Limitations of this study include several assumptions for which we could not find robust evidence, such as rate of re-infections and deaths due to severe strongyloidiasis. However limited, we could anyhow find some papers on which we based our model. Another limitation is that we based some costs for logistics on the budget of a pilot study that is about to start in Ethiopia, so they might not be completely in line with expected expenditure in other countries. The same study is expected to provide further data to analyse the impact of PC with ivermectin targeting SAC. Additional benefits of ivermectin administration (such as impact on scabies and increased efficacy against the other STH) were not quantified but might be considered by endemic countries in the context of other health interventions deemed relevant. Finally, here we do not measure the impact of possible additional interventions, such as the water, sanitation and hygiene (WASH) practices, which can further contribute to reduce the prevalence of STH [27] and are indeed recommended by the WHO [3]. While we support the integration of PC for STH with WASH, the evaluation of its impact was out of the scope of the present study.

## Conclusions

Both PC strategies lead to a dramatic reduction in the prevalence of infection compared to the current situation (no treatment). Strategy B lead to a larger number of adverted deaths than strategy C, but related costs were lower for the latter strategy. An additional aspect that should be considered is that, at the moment, in almost all the areas endemic for strongyloidiasis, are in place school deworming programmes distributing benzimidazoles for the control of STH [3]. Adding ivermectin to this existing school benzimidazole distribution platform would allow to further reduce ivermectin distribution cost for SAC. We believe that this work can provide useful data to countries that wish to implement control strategies for *S. stercoralis*. While PC on the whole population shows a stronger impact on the reduction in the absolute number of infected people and deaths, PC targeting SAC can advert a death with a lower cost. Prevalence of 15–20% or more might be recommended as threshold to recommend the implementation of PC with ivermectin, in consideration of a balance between costs and effectiveness of the intervention.

## Data Availability

The datasets supporting the conclusions of this article are included within the article.

## Abbreviations

STH: Soil-transmitted helminth
WHO: World Health Organization
NTD: Neglected tropical diseases
PC: Preventive chemotherapy
SAC: School-age children
ICER: Incremental cost-effectiveness ratio
USD: United States Dollar
WASH: Water, sanitation and hygiene
